# Magnetic Properties and Carrier Transport of Ir_0.9_Mn_1−x_Sn_1.1+x_

**DOI:** 10.3390/ma12020283

**Published:** 2019-01-16

**Authors:** Diangang Pan, Lu Li, Junyu Yang, Hong Chang

**Affiliations:** School of Physical Science and Technology, Inner Mongolia University, Hohhot 010021, China; pdgnews@163.com (D.P.); m15848161806@163.com (L.L.); maitianyjy@163.com (J.Y.)

**Keywords:** half-Heusler, magnetoresistance, magnetic properties

## Abstract

The nonstoichiometric Ir_0.9_Mn_1−x_Sn_1.1+x_ (x = 0.1, 0.05, and −0.05) are crystallized in half-Heusler alloys. The magnetic transition is observed at 77 K for *x* = 1.05, and it decreases with the decreasing Mn content. It is proven to be a ferromagnetic transition with a nonlinear magnetic moment alignment, as the magnetization is not saturated at 70 kOe. The different magnetic behavior than a typical ferromagnet (FM is due to the Ir ions with strong spin orbital coupling (SOC). The different hysteresis loops reflect that the ionic distribution is not completely homogeneous. The high coercivity observed in the cubic compound is due to the strong single-ion anisotropy of the Ir ions. A metallic-semiconducting transition at 130 K is observed in Ir_0.9_Mn_1.05_Sn_1.05_. A negative magnetoresistance is observed at 2 K and 14 T with the value as −2.6%.

## 1. Introduction

Half-Heusler alloys have attracted a wealth of attention with a variety of interesting properties as magnetic shape memory alloy, thermoelectric effect, magnetocaloric materials, half-metallic materials, and topological insulating states of matter [1,2,3,4,5,6,7]. The half-Heusler alloys crystallize in the noncentrosymmetric cubic structure with the space group *F*-43*m*, and all the atoms possess the same site symmetry -43*m*. Recently, the half-Heusler alloys composed of a heavy transition metal, a light transition metal or rare earth, and a main-group element, such as Bi, Sb, and Sn, have attracted a lot of attention, because some of them have been observed or anticipated as Dirac and Weyl semimetal exhibiting topologically protected Fermi-arc surface [8,9]. In the Ir and Mn containing half-Heusler alloys, IrMnSb is FM with the Curie temperature at 290 K [10]. IrMnAl is FM with a very high Curie temperature above 400 K [10]. In AuMnSb, Sb can be partly substituted by Sn [11]. AuMnSn is FM with a high Curie temperature at 570 K [12]. All these half-Heusler alloys are an interesting subject in the search for topological materials with magnetic properties. As far as IrMnSn is concerned, the magnetic property of Ir_1.07_Mn_1.07_Sn_0.86_ was reported before, and also the enthalpy was done [13,14,15]. Ir_1.07_Mn_1.07_Sn_0.86_ is a half-Heusler compound with 22 electrons. It is a localized moment ferromagnet. The 22 electrons divide themselves into 13 in the majority spin and 9 in the minority spin direction, resulting in a semiconducting gap (half-metallic behavior). A change in the valence electron concentration should strongly affect the properties of the half-Heusler alloy. Ir_0.9_Mn_1−x_Sn_1.1+x_ is close to the 18-valence-electron compounds as a closed shell species. In this paper, we report on the magnetic and electric transport properties of the nonstoichiometric Ir_0.9_Mn_1−x_Sn_1.1+x_ half-Heusler alloy. The magnetic behaviors are different from what were previously reported. It indicates that the composition has a big impact on the magnetic properties. As the topological compounds’ electronic state at the Fermi surface is sensitive to the external stimulus, especially the magnetic field, the present study may give a clue for the future study on topological materials.

## 2. Experiments

All the Ir_0.9_Mn_1−x_Sn_1.1+x_ (x = 0.1, 0.05, and −0.05) alloys were synthesized by the solid state reaction. Mn, Sn, and Ir powders were bought from Alfa Aesar company (Haverhill, MA, USA) with a purity higher than 99.99%. The starting powders were weighted as the nominal composition, and mixed together with a mortar and pedlar. Then, the initial materials were put into an alumina crucible and sealed in a quartz tube with the vacuum about 5 Pa. Afterwards, they were sintered at 920 °C for 50 h and quenched in cold water. The phase characterization was carried out with powder X-ray diffraction (XRD) using an X’pert pro PANAlytical diffractometer with Cu-Kα radiation (Malvern, UK). The compositions were detected by the energy dispersion X-ray spectra (EDX) on Hitachi S-4500II field emission SEM (Tokyo, Japan). The magnetic properties were measured on SQUID from 2 K to 390 K, and the electrical transport was studied with a standard 4-point DC technique and performed on magnetic property measurement system (MPMS, Quantum Design, Inc., San Diego, CA, USA) from 1.85 K to 390 K.

## 3. Results and Discussion

**Figure 1:**
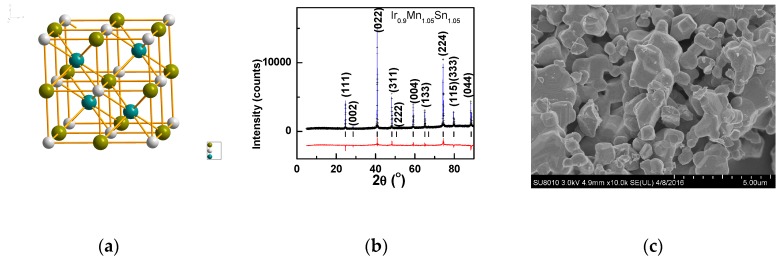
(**a**) The unit cell of the half-Heusler alloy; (**b**) the XRD pattern of Ir_0.9_Mn_1.05_Sn_1.05_ with the black cross as the experimental data, the blue line as the fitted pattern, the red pattern at the bottom as the difference between the experimental and the calculated pattern, and the black ticks in the middle as the Bragg positions; and (**c**) SEM image of Ir_0.9_Mn_1.05_Sn_1.05_.

In the half-Heusler alloy, if all the bonding states were occupied by 18 valence electrons and all the antibonding states were empty, it formed a stable compound. A few extra electrons slightly decreased the stability by entering into the antibonding states [16,17]. Taking the above rules into consideration, Ir_0.9_Mn_1−x_Sn_1.1+x_ with about 18 electrons may be more stable than the stoichiometric IrMnSn with 20 electrons. Ir_0.9_Mn_1−x_Sn_1.1+x_ (x = 0.1, 0.05, and −0.05) with single phase was synthesized. One unit cell of Ir_0.9_Mn_1−x_Sn_1.1+x_ is shown in Figure 1a, with the space group as *F*-43*m*. From the XRD pattern, it was impossible to tell the difference between the half-Heusler phase and the Heusler phase. However, as the composition was close to 1:1:1, as confirmed by the EDX and the XRD refinement, and no impurity was observed, Ir_0.9_Mn_1−x_Sn_1.1+x_ (x = 0.1, 0.05, and −0.05) should crystallize in the half-Heusler phase. Figure 1b shows the XRD pattern of Ir_0.9_Mn_1.05_Sn_1.05_ as a representative. In the refinement of the XRD patterns, Ir^4+^ ions were at the 4b site, and Mn ions occupied the rest of the 4b site. Most of the Mn ions were at the 4a site, and the rest of them entered the 4c site. Similarly, most of the Sn ions are at the 4c site, and the rest of them enter the 4a site, as listed in Table 1. The lattice expanded with the increasing Sn content (or the decreasing Mn), as the ionic radius of Sn^4+^ was larger than that of Mn^4+^. The particle size is in the order of 1–3 μm, as shown in Figure 1c. 

**Figure 2:**
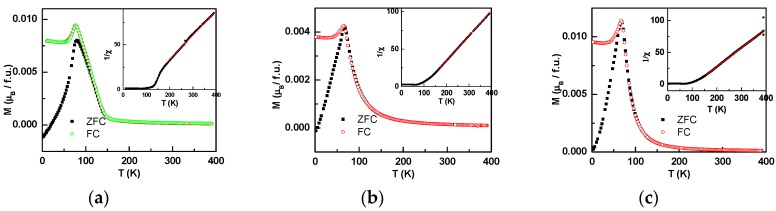
The magnetization versus the temperature of Ir_0.9_Mn_1.05_Sn_1.05_ (**a**), Ir_0.9_Mn_0.95_Sn_1.15_ (**b**), and Ir_0.9_Mn_0.9_Sn_1.2_ (**c**), and the inset is the corresponding inverse susceptibility versus the temperature with the red line as the fitting curve to the Curie-Weiss law.

Figure 2a–c shows the variations in the magnetization with the temperature for Ir_0.9_Mn_1−x_Sn_1.1+x_ (x = 0.1, 0.05, and −0.05) at the zero-field cooling (ZFC) and the field cooling (FC) mode measured at 0.5 kOe, respectively. The ZFC magnetization of Ir_0.9_Mn_1.05_Sn_1.05_ decreased sharply as the temperature was below the peak temperature 77 K, which was generally related to an antiferromagnetic (AFM) transition. However, the flat FC magnetization below 60 K indicated that it was more than an AFM transition. The DC susceptibility with the temperature higher than the transition temperature is fitted with the Curie–Weiss law, $\chi =\frac{dM}{dH}=\frac{C}{T-\theta }$, as shown in the inset of Figure 2a for *x* = 1.05. The linear dependence of 1/χ on T excluded the possibility of a ferrimagnetic (FiM) phase, since for a FiM phase, the dependence of 1/χ on T should be curved. The obtained positive Weiss temperature, θ = 106 K, indicated that the magnetic moment alignment below *T_c_* was FM, and the effective magnetic moment obtained was 3.86 *μ_B_* per formula unit. In Ir_0.9_Mn_1−x_Sn_1.1+x_, Mn was not the only magnetic ion, and the Ir ion should not be ignored. The spin orbital coupling (SOC) was very important for the Ir^4+^ ion, as SOC was proportional to Z^4^ and Ir had a large atomic number Z. The local spin **S** and orbital **L** moments tend to bind into a total angular momentum **J** = **S** + **L** by the strong SOC, and **J** obeyed the same spin-commutation rules. Considering that Ir^4+^ had 5 electrons, one hole with an effective orbital momentum *l* = 1 and a spin *s* = 1/2 was formed [17]. Such an Ir^4+^ ion possessed strong single-ion anisotropy, and it would greatly influence the magnetic properties including the high coercivity in Ir_0.9_Mn_1−x_Sn_1.1+x_. As the coupling among the Ir ions was AFM due to the half-filled localized 5d orbitals, it was supposed that the AFM-like transition was related to the Ir ions.

In fact, it was reported that IrMnSn was FM with the Curie temperature *T_c_* = 204 K [13]. The *T_c_* difference between the present study, below 77 K, and what was reported before is large. It indicates that the magnetic interaction is highly sensitive to the chemical composition. The interatomic distances were calculated based on the atomic positions obtained from refining the XRD patterns. In the nonstoichiometric Ir_0.9_Mn_1−x_Sn_1.1+x_, the interatomic *Mn-Mn* distance varied from 2.7051 Å to 3.1236 Å. The short *Mn-Mn* distance below 3 Å was in favor of the AFM interaction, while that above 3 Å was in favor of FM. Since the magnetic interactions among Mn ions are either AFM or FM, it leads to the sensitivity of the magnetic phase to the composition.

Figure 2b,c show the thermal magnetization of Ir_0.9_Mn_0.95_Sn_1.15_ and Ir_0.9_Mn_0.9_Sn_1.2_ at the FC and the ZFC modes measured at 0.5 kOe. Ir_0.9_Mn_0.95_Sn_1.15_ and Ir_0.9_Mn_0.9_Sn_1.2_ had the magnetic transition peaks at 69 K and 67 K, respectively. The Weiss temperatures obtained by fitting the inverse susceptibility with the Curie-Weiss law were 97 K and 79 K for *x* = 0.95 and 0.9. The decrease of the Weiss temperature indicates that the strength of the FM interaction decreases with the decreasing Mn content. The effective magnetic moment was 3.62 *μ_B_/f.u.* and 4.07 *μ_B_/f.u.* for Ir_0.9_Mn_0.95_Sn_1.15_ and Ir_0.9_Mn_0.9_Sn_1.2_, respectively.

**Figure 3:**
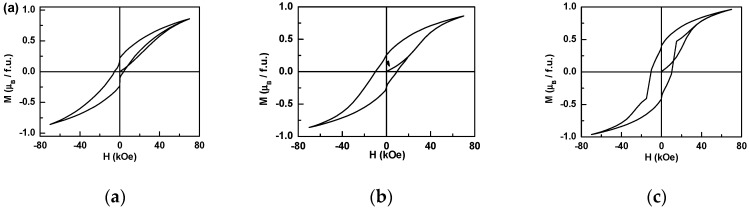
The hysteresis loop of the magnetization of Ir_0.9_Mn_1.05_Sn_1.05_, (**a**), Ir_0.9_Mn_0.95_Sn_1.15_ (**b**), and Ir_0.9_Mn_0.9_Sn_1.2_ (**c**).

As shown in Figure 3a, the hysteresis loop of Ir_0.9_Mn_1.05_Sn_1.05_ measured at 2 K supports that it is an FM phase. A high coercivity as 5 kOe was observed. Since the magnetocrystal anisotropy was very low in the face-centered cubic compound, the single ion anisotropy of the Ir^4+^ ion with a strong SOC contributed to the high coercivity. As the magnetic field changed directions, the absolute value of the magnetization abruptly dropped. It indicated that the magnetic phases were not completely homogeneous. As the *Mn* magnetic moment was not pinned by the Ir ion, the anisotropy was very low and induced a sharp drop as the applied field reversed its direction. The magnetization at 70 kOe was only 0.86 *μ_B_/f.u.*, and it still kept increasing even at 70 kOe. It indicated that the alignment of the magnetic moment is nonlinear.

Figure 3b,c show the initial magnetizations and the hysteresis loops of Ir_0.9_Mn_0.95_Sn_1.15_ and Ir_0.9_Mn_0.9_Sn_1.2_. Both of them have the coercivity as 10 kOe. Similar magnetization jumps were observed at 0^−^ T and 0^+^ T in both Ir_0.9_Mn_0.95_Sn_1.15_ and Ir_0.9_Mn_0.9_Sn_1.2_, but the scale was much smaller than that in Ir_0.9_Mn_1.05_Sn_1.05_. The magnetization at 70 kOe was 0.96 *μ_B_/f.u.* and 0.85 *μ_B_/f.u.* for Ir_0.9_Mn_0.95_Sn_1.15_ and Ir_0.9_Mn_0.9_Sn_1.2_, respectively, and it still increased at 70 kOe. Furthermore, the hysteresis loop of Ir_0.9_Mn_0.9_Sn_1.2_ had a jump at 1.5 T, which was not observed in the other two compounds. It was owed to the pinning effect at the domain boundaries. The different contours of the hysteresis loops indicated that the magnetic phases were not homogeneous, and the composition and ionic distribution had a big impact on the magnetic properties.

**Figure 4:**
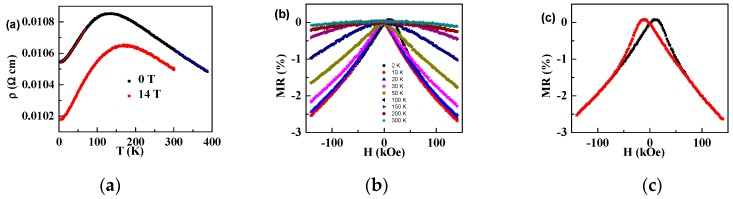
(**a**) The resistance versus the temperature of Ir_0.9_Mn_1.05_Sn_1.05_, at 0 T and 14 T, with the red line as the fitting line with the metallic transport *ρ* = *ρ*_0_ + *BT*^2^; (**b**) magnetoresistance (MR) versus the applied magnetic field of Ir_0.9_Mn_1.05_Sn_1.05_ at 2 K, 10 K, 20 K, 30 K, 50 K, 100 K, 150 K, 200 K, and 300 K; and (**c**) MR of Ir_0.9_Mn_1.05_Sn_1.05_ at 2 K with the hysteretic butterfly shape.

The variations of the resistivity versus the temperature at 0 T (black solid square) and 14 T (red solid circle) are shown in Figure 4a. Below the peak temperature at around 130 K, the resistivity increased with the temperature, i.e., the thermal resistivity behaved like a metal. Above the peak temperature, the resistivity decreased, and had a semiconducting behavior. In the temperature range from 30 K to 195 K, the resistivity at 0 T is fitted with the metallic behavior as *ρ* = *A* + *CT*^2^, shown as the red line in Figure 4a. As generally accepted, the 18-electron configuration of the half-Heusler alloy gave a full bonding state, which led to a semiconducting state, while either 17- or 19-electron configuration decreased the stability and led to a metallic state. As the resistivity at 14 T is lower than that at 0 T, as shown in Figure 4a, the aligned magnetic moments have an impact on the resistivity. In order to further investigate the magnetoresistance (MR), MR versus the magnetic field is measured at different temperatures, as shown in Figure 4b. $MR=\frac{M\left(H\right)-M\left(0\right)}{M\left(0\right)}\times 100\%$ is negative and has a butterfly shape at 2 K, as shown in Figure 4c. It was −2.6% at 2 K and 14 T, and it was −0.1% at 300 K. The butterfly shape characterized that the MR was influenced by the magnetization tunneling across the domain boundaries. It indicated that the MR originated from the suppression of the spin-disorder scattering.

## 4. Conclusions

Ir_0.9_Mn_1−x_Sn_1.1+x_ crystallized in half-Heusler alloys with the space group *F*-43*m*. The Ir^4+^ ion with a strong SOC contributed to the high coercivity, which was generally not observed in the face centered cubic compound. However, as the magnetic phases were not completely homogeneous, the magnetic moments of those Mn ions, not influenced by the Ir, dropped sharply as the magnetic field changed its direction. The inhomogeneous magnetic interactions were also confirmed by the different contours of the hysteresis loops of Ir_0.9_Mn_1−x_Sn_1.1+x_. The magnetic moment alignment was nonlinear as the magnetization at 70 kOe still kept increasing. A metallic-semiconducting transition at 130 K and a negative MR were observed in Ir_0.9_Mn_1.05_Sn_1.05_. The spin-disorder scattering was suppressed as the magnetic moment was aligned, and it resulted in a negative MR.

## Figures and Tables

**Table 1:** The lattice parameters and site occupancies of Ir_0.9_Mn_1−x_Sn_1.1+x_.

x	Ir_0.9_Mn_0.9_Sn_1.2_	Ir_0.9_Mn_0.95_Sn_1.15_	Ir_0.9_Mn_1.05_Sn_1.05_
a (Å)	6.2658 (4)	6.2610 (4)	6.2472 (1)
Ir (4b) Occ.	0.88	0.90	0.89
Mn (4b) Occ.	0.12	0.10	0.11
Mn (4a) Occ.	0.65	0.67	0.68
Sn (4b) Occ.	0.35	0.33	0.32
Sn (4c) Occ.	0.88	0.83	0.75
Mn (4c) Occ.	0.12	0.17	0.25
